# Sampling effects in quantum mechanical/molecular mechanics trajectory surface hopping non-adiabatic dynamics

**DOI:** 10.1098/rsta.2020.0381

**Published:** 2022-05-16

**Authors:** Davide Avagliano, Emilio Lorini, Leticia González

**Affiliations:** ^1^ Faculty of Chemistry, Institute of Theoretical Chemistry, University of Vienna, Währinger Straße 17, A-1180 Vienna, Austria; ^2^ Vienna Research Platform on Accelerating Photoreaction Discovery, University of Vienna, Währinger Straße 17, A-1180 Vienna, Austria

**Keywords:** surface hopping, QM/MM, initial conditions

## Abstract

The impact of different initial conditions in non-adiabatic trajectory surface hopping dynamics within a hybrid quantum mechanical/molecular mechanics scheme is investigated. The influence of a quantum sampling, based on a Wigner distribution, a fully thermal sampling, based on classical molecular dynamics, and a quantum sampled system, but thermally equilibrated with the environment, is investigated on the relaxation dynamics of solvated fulvene after light irradiation. We find that the decay from the first singlet excited state to the ground state shows high dependency on the initial condition and simulation parameters. The three sampling methods lead to different distributions of initial geometries and momenta, which then affect the fate of the excited state dynamics. We evaluated both the effect of sampling geometries and momenta, analysing how the ultrafast decay of fulvene changes accordingly. The results are expected to be of interest to decide how to initialize non-adiabatic dynamics in the presence of the environment.

This article is part of the theme issue ‘Chemistry without the Born–Oppenheimer approximation’.

## 1. Introduction

Trajectory surface hopping (TSH) is a widely used method to simulate excited states non-adiabatic dynamics [1,2]. The method is based on the propagation of an ensemble of independent trajectories, which evolve individually with the nuclei propagated following Newton's Law of Motion on potential energy surfaces (PESs) calculated on-the-fly. When a trajectory is in the proximity of a conical intersection (CI) [3], the non-adiabatic effects arising from the coupling among nuclear and electronic motions are approximated so that either a switch takes place between two electronic states through instantaneous hops or the trajectory keeps propagating on the same surface. Through a statistically reasonable ensemble of trajectories, it is possible to mimic the behaviour of a splitting wave packet [4]. Despite its approximations, surface hopping has become very popular as it allows the description of the excited states dynamics of many systems, from small to large, depending on the underlying expense involved in the method employed to calculate the PES on-the-fly. Here one can choose from the large variety of quantum mechanical (QM) methods [5] or resort to semi-empirical approximations [6]. Yet, when solvent and environmental effects need to be considered, further approximations are required [7]. One smart way to consider the interaction between a molecule and its environment is to treat them at different levels of theory. In its simplest form, the chromophore can be treated with QM at the high level of theory, while the energy of other parts of the system and the solvent molecules can be calculated by means of classical force fields. This approach is known as quantum mechanical/molecular mechanics (QM/MM) [8], and it is efficiently used to include environmental effects in ground and electronically excited states simulations [9].

One important ingredient in TSH simulations is the generation of initial conditions, i.e. the sampling of the distribution of positions and momenta. Interestingly, it has been shown that this can influence the results of the nuclear dynamics obtained [10,11]. One approach to generate initial conditions is to sample thermally the configurational space, where the vibrational energy for each normal mode is given by *k_b_T*, obtained with a classical or *ab initio* molecular dynamics in the electronic ground state. This approach neglects quantum effects, like the zero-point energy (ZPE), which is instead included if a quantum sampling of the phase space is employed. Here, a common approach is to calculate a Wigner distribution of probability densities of positions and momenta for each normal mode [12]. Additionally, several approaches combining [13] or improving [14] the aforementioned approaches to obtain position and velocities distributions have been lately developed. While these different ways of sampling have been tested and compared in the past for surface hopping dynamics in the gas phase [10], the situation becomes more complex within a QM/MM scheme, where the chromophore interacts with the environment, and it is in thermal equilibrium with it [15]. In this work, we want to investigate the effect of different methodologies for sampling the initial conditions in TSH trajectories but applied to a QM/MM framework. As a test case, we choose fulvene, which undergoes ultrafast dynamics and has been well studied in the past in the gas phase [16–19]. After population of the first electronically excited state, fulvene can relax to the electronic ground state through two CIs, a peaked and a sloped one, which are associated with the torsion and the stretching of the C=C alkene bond, respectively. The sloped CI allows for a reflection of the population, which comes back to the S_1_. For this reason, fulvene has been recently proposed as a molecular model [20] to describe non-adiabatic processes, in particular reflection of population on excited states PES, in analogy to the two-dimensional model proposed by Tully in 1990 [1]. Interestingly, the authors of [20] showed that the decay and reflection through the sloped CI or the relaxation through the peaked one is strongly dependent on the simulation parameters. This motivated us to investigate whether modulating the initial conditions can also change the dynamics of fulvene in water. The non-polarity of the molecule helps us to focus on the dynamical behaviour induced exclusively by the change of the different initial values of the vibrational kinetic energy (KE) and geometries, by avoiding physical (for instance hydrogen bonds) interactions between the chromophore and the solvent throughout the course of the dynamics. We limit the solvent role to electrostatic effects, yet analogous for each set of generated initial conditions. For these calculations, we shall use our recently developed scheme to run QM/MM non-adiabatic dynamics simulations [21] based on SHARC [22,23] and COBRAMM [24,25] software, which provide TSH and the QM/MM implementations, respectively. We will show how changing the initial momenta, either due to manual selection or through energy exchange and equilibration between the QM and the MM region, will lead to very different and interesting results that should be kept in mind when deciding how to sample initial conditions for QM/MM TSH simulations.

## 2 Methods

### (a) Sampling methods

**Figure 1.**
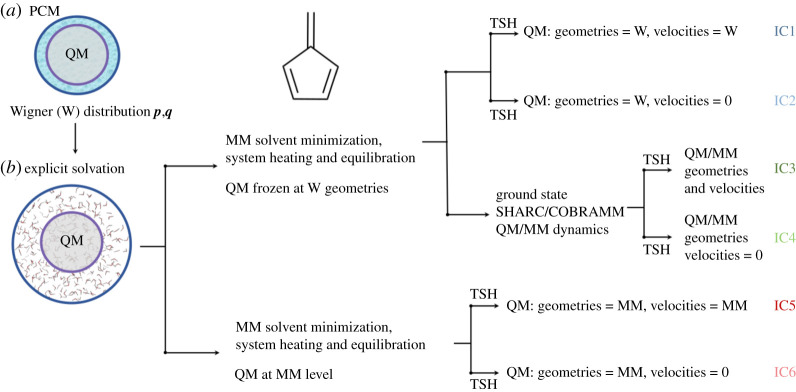
Schematic representation of the generation of six different ensembles of initial conditions (IC1–6) for QM/MM non-adiabatic dynamics. (Online version in colour.)

We shall consider six different sets of ensembles of initial conditions, schematically depicted in figure 1. First, we calculated a Wigner distribution of the QM part to sample the ground state phase space of isolated fulvene. The solvent effects on the distribution are considered by optimizing the geometry and calculating the normal modes including the effect of water by implicit solvation [26] with a polarizable continuum scheme (PCM).

We then solvated each of the geometries obtained with explicit water molecules. We optimized, heated and equilibrated each individual geometry. In a first case, we did these steps keeping the QM part frozen at the Wigner geometries. The first TSH set of trajectories is then initialized from Wigner geometries and velocities, surrounded by MM water molecules (IC1). In [20] it was shown that, in the gas phase, the sloped CIs can be reached more efficiently over the peaked one if the velocities are set to zero. In order to test this effect in water, we initialized a second set of trajectories from Wigner geometries with the velocities set to zero (IC2). Additionally, we took the solvated QM geometries and from the Wigner positions and momenta we ran a ground state post-equilibration at the QM/MM level with the SHARC/COBRAMM scheme during 100 fs. After that, we initialized a third set of trajectories (IC3). As in the previous case, an additional set, where the velocities were set to zero, was also prepared (IC4). Last family of initial conditions was fully thermally sampled. Starting from the solvated molecules, we minimized, heated and equilibrated the system at MM level, including the QM part treated at the force field level. At the end of the equilibration, two sets of trajectories were initialized, one with geometries and velocities obtained at the MM level for the whole system (IC5) and one with geometries at MM level and again velocities set to zero (IC6). Summarizing, we sampled the QM region at a quantum level (IC1 and IC2), with a quantum system, but with the vibration relaxed and equilibrated with the surrounding water molecules (IC3 and IC4) and with a full thermal sampling obtained by classical molecular mechanics (IC5 and IC6).

### (b) Quantum mechanical/molecular mechanics scheme

The QM/MM scheme used in this work follows the recent [19] implementation resulting from interfacing SHARC and COBRAMM. Highlights in the QM/MM implementation include (i) a subtractive scheme for the calculation of the energy and the gradient of the electronic states [8], with the implementation of an electrostatic embedding scheme [27] to account for the polarization of the QM part due to the presence of the surrounding MM environment, included in the adopted Hamiltonian as point charges; (ii) the inclusion in the MM gradient of a state-specific term due to the force induced by the QM region on the point charges; (iii) the QM molecule surrounded by a droplet of homogeneous radius of water molecules and, in order to address the lack of periodical boundary conditions, the external shell of water molecules is kept frozen to furnish a constant potential and keep the droplet stable. Additional information and the full implementation can be found in the original publication [21].

### (c) Computational details

All the QM calculations were performed with the complete active space self-consistent field (CASSCF) [28] using six electrons in six active orbitals, combined with a 6-31(G)d basis set [29], denoted as CASSCF(6,6)/6-31(G)d level of theory. The active orbitals are the 3 π and 3 π* orbitals of the fulvene. These calculations are performed with the OpenMolcas software [30]. The quantum distribution of the electronic ground state of the QM geometries and velocities was obtained at the fulvene optimized geometry where each of the normal modes was sampled with a harmonic quantum Wigner oscillator. The sampling was performed at 300 K to include the possible population of different vibrational states [31] and the effect of the environment was included in the optimization of the molecule and the computation of the normal modes with PCM, [26] as implemented in OpenMolcas. A total amount of 500 geometries was solvated with a box of water molecules, and the system was minimized for 500 cycles with the steepest descent algorithm and for 2000 with the conjugated gradient method. After that, the systems were heated at 300 K in 50 ps, pressure and volume were equilibrated for 100 ps with the temperature kept constant to 300 K with a Nosé–Hoover thermostat [32]. During these steps, the QM part was kept frozen at the initial geometry obtained from the Wigner distribution and, at the end of this set-up, a droplet of 500 solvent molecules was stripped around the centred chromophore. All the MM calculations were carried out with AMBER suite [33].

At this stage, we obtained the six different ensembles of initial conditions for TSH. For each of them, the same TSH parameters (see below) were adopted. We ran 500 independent trajectories from all the 500 Wigner geometries that we solvated. The simulation time was 70 fs, with a nuclear time step of 0.5 fs. Only two states, S_1_ and S_0_, were included. The non-adiabatic coupling was approximated by evaluating the time-derivative coupling through wave function overlaps [34]. The energy-based decoherence correction scheme [35], with a decoherence parameter of 0.1, and an atom masking to exclude the solvent molecules from the velocities rescaling procedures was employed [21].

The water molecules were treated with the flexible force field SPC/Fw [36], as available in AMBER. During the SHARC/COBRAMM dynamics, the 300 molecules of the droplet closest to the QM part were allowed to move, while the most external 200 ones were kept frozen. For IC5 and IC6, the set-up steps were analogous, with the difference that the chromophore was allowed to move under the effect of the classical general AMBER force fields potentials [37] and the TSH was initialized from the last snapshot of the equilibration procedure.

## 3. Results and discussion

**Figure 2.**
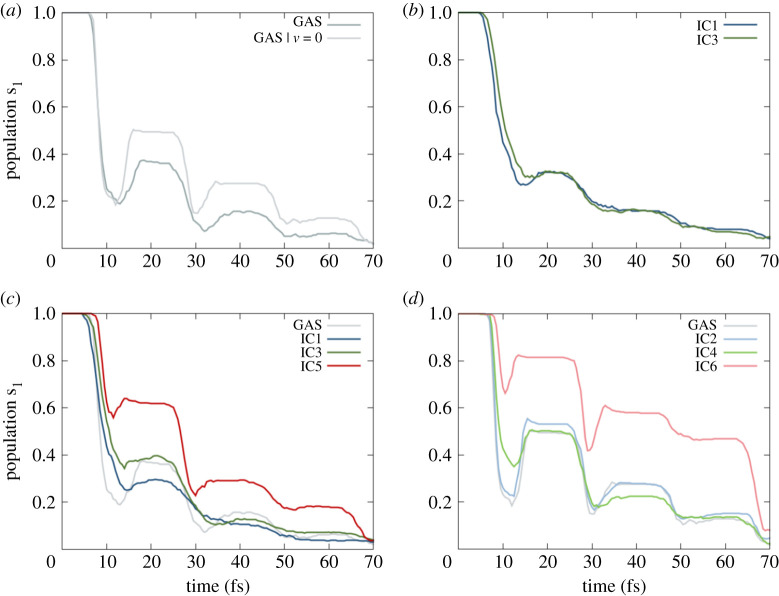
Time-resolved population of the first excited state (S_1_) for the different ensemble of trajectories: (*a*) population of 250 gas phase trajectories starting from initial velocities obtained from a Wigner distribution (GAS) or set to zero (GAS | *v* = 0); (*b*) population relative to 500 QM/MM trajectories, with all the 500 water molecules kept frozen, for IC1 and IC3; (*c*) population relative to 500 QM/MM trajectories with 300 water molecules allowed to move and the most external 200 ones frozen for IC1, IC3 and IC5 and (*d*) analogous population obtained from IC2, IC4 and IC6. (Online version in colour.)

Theoretical studies in gas phase [17–19] showed that, after populating the first electronic excited state, fulvene undergoes a double-decay relaxation within the first tens of femtoseconds. Interestingly, the preference for one or another competitive decay pathway can be strongly influenced by the initial conditions and the simulation parameters chosen to initialize and propagate the excited states dynamics [20]. In [20], the authors showed how the reflective CI, connected to the stretching of the C=CH_2_ bond, is easily reached in TSH simulations when the velocities of the initial geometries were set to zero. In our reference gas phase TSH dynamics (figure 2*a*), we observed the decay to S_0_ within the first 10 fs and the population reflected to S_1_ within in the first 30 fs in both dynamics—initializing the trajectories from Wigner velocities or velocities set to zero—but with a notably increased reflection in the second case. Note that our simulations employ a different number of trajectories than [20] and that we use 250 different initial geometries. Additionally, we extended the dynamics up to 70 fs, including two more reflection cycles.

Having obtained a general picture in gas phase, we can now move to study the effect of the QM/MM set-ups on the dynamics. We first proceed to evaluate the pure electronic effect of the presence of the solvent. For this purpose, we first ran TSH dynamics with IC1 and IC3, but keeping frozen all the solvent molecules and letting the fulvene move in a constant static potential given by the same point charges distribution (figure 2*b*). Note that, in this example, in the case of IC3, both in the ground state equilibration and in the TSH the water molecules are kept frozen. Regardless of whether we initialize the dynamics directly from the Wigner position and momenta (IC1) or we take the same initial conditions and first ran a ground state dynamics (IC3), the S_1_ population profiles decay very similarly. In both cases, the decay time to S_0_ does not change with respect to the gas phase, but the inversion of population within the first 30 fs is reduced and fully disappears in the next two reflection cycles. A slight difference between the dynamics based on IC1 and IC3 is present in the population around 15 fs. While IC1 shows a bit of inversion of the population trend, IC3 shows a fully flat population. However, both decays continue flatly and constant without showing any reflection from 25 to 70 fs in the presence of the electrostatic potential induced by the water. We had nevertheless expected that including the interaction of the QM part with a shell of mobile explicit water molecules would affect the TSH dynamics. Indeed, the equilibration of the vibrational modes of the QM molecule includes the interaction with the solvent molecule, and the thermal equilibration of the whole system accounts for the equilibration of the water degrees of freedom. In this sense, the choice of a flexible force field is fundamental, allowing the stretching of the O-H bonds of the solvent molecules, which would otherwise be constrained in the case of a rigid model, such as the common TIP3P choice [38]. Such constraint would remove vibrational modes of the solvent molecules from the thermal equilibration, altering the changes in vibrational energy distribution of the QM part, and we would not include the interactions of all the solvent normal modes on the position and momenta distributions of fulvene.

**Figure 3.**
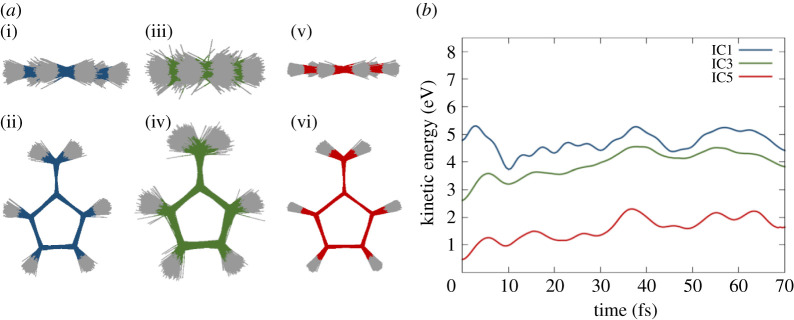
(*a*) Cluster of 500 initial geometries obtained by different sampling methods: (i, ii) IC1, (iii, iv) IC3 and (v, vi) IC5; (*b*) averaged KE of the QM region during the TSH dynamics for IC1, IC3 and IC5. (Online version in colour.)

In addition to the general effect of the explicit mobile waters, we expected differences in the dynamics according to the different sets of distributions of IC. Indeed, the ensemble of geometries obtained with the three sampling methods (quantum, quantum plus thermal equilibration, fully thermal) came out to be rather different from each other (figure 3*a*) and so is the KE of the associated QM part (figure 3*b*). The Wigner distribution is obtained for the normal modes at the optimized geometry, but once the molecule is allowed to relax from this frozen geometry in the presence of the water, it displaces its normal modes over the conformational space (figure 3*a*). At the same time, it gets colder due to the thermal equilibration with the surrounding solvent molecules, as can be seen by the decrease of the averaged initial KE of the QM part for IC3 that is halved with respect of IC1 (figure 3*b*). By contrast, the full classical thermal sampling produces a sampling more limited in the conformational space (figure 3*a*). This sampling method of IC5 produces a much colder QM part. Indeed, in IC5, fulvene increase its KE during the TSH dynamics while interacting with the solvent molecules (figure 3*b*).

The differences in initial KE among IC1, IC3 and IC5 are reflected in their S_1_ population decays (figure 2). Compared to IC1 driven dynamics, that from IC3 leads to 10% more population trapped in the S_1_ state between 10 and 30 fs (figure 2*c*). This difference in population, which was absent in the case of frozen water molecules, it can be ascribed to the equilibration with the solvent along the ground state QM/MM dynamics. Nonetheless, in both IC1 and IC3 cases, the population profile is flat and does not show the same reflection found in the gas phase. After 30 fs, in both cases the S_1_ population decays to the ground state similarly along the torsional path, without showing any reflection. The situation drastically changes for IC5, where the chromophore was treated classically (figure 2*c*). Here, after 10 fs, only 40% of the population is transferred to S_0_, in contrast with the almost 80% and 60% of IC1 and IC3, respectively. The reflection to S_1_ is now more pronounced, making the S_1_ population between 10 and 30 fs approximately double than in case of IC1. Two reflection cycles are clearly present during the rest of the dynamics. The lower KE, the absence of ZPE and the harmonic classical potential used for this sampling, they all make the relaxation less pronounced and passing through the sloped CI. In order to exclude the possibility that such differences in population are not an artefact of the decoherence correction scheme used, where the damping of the electronic coefficient is based on the KE value of the chromophore, we have re-ran the same trajectories for IC1 with different decoherence parameters (instead of default 0.1, set to 0.3 and 0.7 in this test). We found (results not shown) that the population profile remains the same and do not converge to the one of IC3 or IC5, thus excluding unphysical artefacts due to the decoherence correction scheme employed.

**Figure 4.**
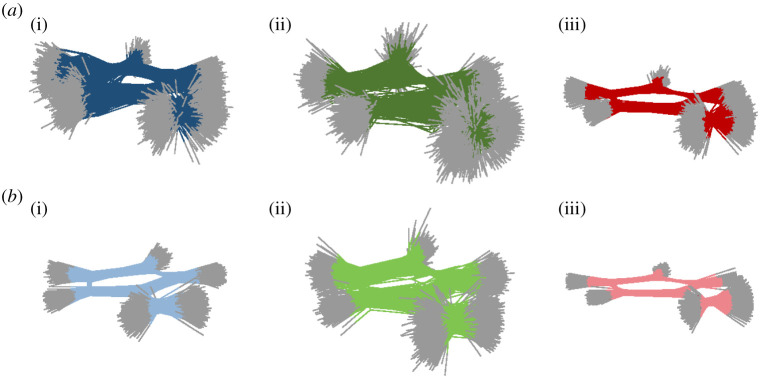
Cluster of hopping geometries from S_1_ to S_0_ around 10 fs for a set of trajectories starting from (*a*) IC1 (i), IC3 (ii) and IC5 (iii) and starting from (*b*) set of trajectories with null initial momenta IC2 (i), IC4 (ii) and IC6 (iii). (Online version in colour.)

Regarding fulvene dynamics in the gas phase, it was shown that setting the initial velocities to zero made easier to reach the sloped CI, leading to a strong reflection of the population in the first tens of fs. Now, we want to investigate whether this effect is also present within the QM/MM set-ups (figure 2*d*). Setting the momenta always to zero, the differences among IC2, IC4 and IC6 should mainly reflect the differences in the sampling on the conformational space, helping to disentangle and discriminate the effect of the sampled geometries or velocities. As anticipated, in all IC2, IC4 and IC6-based dynamics, the sloped CI is now easily reached (figure 2*d*) and the system shows high degree of reflection to S_1_ once S_0_ is populated. IC2 leads basically to the same dynamics as in gas phase, with the same behaviour along the whole dynamics and the same degree of reflection. However, IC4 triggers a strong reflection in the 10–30 fs region, while the S_1_ population is flat when using IC3. In all IC2, IC4 and IC6, the decay along the sloped CI is followed also during the rest of the dynamics, with the respective proportion among the three reflection cycles. IC6 induces an enhancement of the S_1_ population in the region of the first reflection and, as was the case for IC5, only a small fraction of the population decays to the ground state within first 10 fs. A clear second reflection is present between 30 and 50 fs and half of the population is still in the first excited states along the first 60 fs. The full classical sampling of the QM geometries produces a slower relaxation from the S_1_ and a prevalence of the relaxation through the sloped CI over the peaked one, as can be seen be similar features present in both IC5 and IC6 driven S_1_ population profiles. In both, fully quantum sampling and quantum followed by thermal relaxation, the presence of the environment leads the dynamics through the peaked CI, with no substantial differences when the water molecules are frozen. This is confirmed by clustering the hopping geometries for the first S_1_ -> S_0_ transition (figure 4) and comparing with the averaged values of the C=CH_2_ and C-C=C-H torsion (table 1).

**Table 1.** Averaged values of the C=CH_2_ stretching and the C-C=C-H torsion for all the initial conditions sets at time step *t* = 0 (initial) and the hopping geometry of the first S_1_ → S_0_ relaxation within 10 fs.

	C=CH_2_ (Å)		C-C=C-H (°)	
	initial	S_1_→S_0_ hop	initial	S_1_→S_0_ hop
IC1	1.36	1.56	9.05	22.52
IC2		1.59		11.01
IC3	1.35	1.59	13.06	24.82
IC4		1.59		14.77
IC5	1.35	1.58	5.25	15.95
IC6		1.59		9.72

The hopping geometries result to be very different between IC1, IC3 and IC5, with only the last one keeping the original planarity. The value of the averaged torsional for the S1-S0 hopping shows how, the more the molecule is bent, the more likely the path through the peaked CI is (table 1). Indeed, the C=CH_2_ stretching value does not change for any of the cluster of the hopping geometries, while the difference in initial momenta, consequence of the different samplings, influences the motion of the torsion and the energy of that mode. Interestingly, the equilibration with the solvent in the ground state of the Wigner geometries, on the one hand side, makes the chromophore colder, and thus shows a decrease of KE (figure 3*b*), but on the other side redistributes the vibrational energy and allows stronger torsion. Setting the initial velocities to zero keeps the orientation of the hopping geometries within the first 10 fs as the initial one, resulting in IC2 and IC6 still very planar, but IC4 distorted by the previous interaction with the solvent molecules during the ground state dynamics (figure 4*b*).

In summary, we have shown that changing the sampling method can strongly influence the geometrical distribution, the energetics and thus the evolution of the trajectories. Despite such an awareness is present in gas phase excited state dynamics [10], attention is needed when explicit solvent and environmental interactions are considered. This is particularly true when two different levels of theory are combined, such as in the multi-scale QM/MM schemes. Often, the high number of degrees of freedom to be described and the high flexibility of the chromophore force a thermal sampling, typically based on classical molecular dynamics simulations. A full thermal sampling is ran using approximated harmonic potentials, it misses the ZPE and produces a colder system, that prevents high energy modes to be properly sampled. The latter problem can also be tackled by a local reheating [39], but such an approach still lacks quantum effects in the phase space distributions. A full quantum sampling, obtained for example with a Wigner probability distribution, is a double-edged knife, as it considers quantum effects like the ZPE, but often delivers too high energy modes, a distribution limited to a single minimum geometry and the impossibility of explicitly account for the interaction with solvent molecules. An interesting alternative approach is the one we adopted in the IC3 set-up. By employing a QM/MM ground state molecular dynamics, the Wigner geometries and momenta of the chromophore are equilibrated with the solvent degrees of freedom; in this way, IC3 allows to keep the information relative to the ZPE, to redistribute vibrational energy along the lower energy vibrational modes and to account for a direct interaction and polarization of the explicit solvent molecules. Therefore, we believe that the approach employed in IC3 is a reasonable compromise out of the different sampling methods and the most suitable for QM/MM TSH set-ups. At the same time, we are aware these sampling methods are not the only possibly way to generate initial condition for semi-classical non-adiabatic dynamics. Mixed classical-quantum methods have been highly promising to describe the shape of the UV absorption of dyes [40,41] or more complicated systems as transition metal complexes [39] or biological structures [42] in explicit solvent environments. It would be interesting to compare their applicability along non-adiabatic dynamics simulations. Further, sampling methods that include laser fields in order to better reproduce wavepackets formed by laser experiments have been also developed [43], and it would be interesting to see their extension to a QM/MM framework.

## 4. Conclusion

In this work, we applied our recently developed approach for running QM/MM TSH simulations, based on the interface of SHARC and COBRAMM codes, to study an apparently simple, yet delicate aspect of non-adiabatic dynamics: the generation of initial conditions. If the complexity of this task is non-trivial in gas phase calculations, we show here that even more care is needed when solvent degrees of freedom and their interaction with the chromophore are also included in the sampling of the QM part within hybrid QM/MM approaches. We compared the dynamical effects of employing (i) a quantum approach, based on Wigner sampling and successive solvent relaxation, (ii) a quantum approach but thermally relaxed, with the Wigner position and momenta equilibrated with the surrounding mobile environment, and (iii) a fully thermal sampling, completely relying on classical force field potentials. The three sampling approaches show important differences given by the initial values chosen to initialize the dynamics, as evident in the S_1_ relaxation decay of a proof-of-concept molecule, fulvene. The first S_1_ → S_0_ decay occurs for all the cases within the first 10 fs, but the amount of population transferred to the ground state changes according to different sets of geometries and initial KE (IC5 > IC3 > IC1). The higher initial momenta the QM part has, the more prompt is to decay to S_0_ through a peaked CI, while a lower amount of KE induces more the decay through the sloped CI connected to the C=CH_2_, with annexed reflection and population transfer back to the S_1_. As in gas phase, this reflection is enhanced by setting the initial momenta to zero, but with clear differences in the amount of population transferred according to the initial geometry sampled (IC6 > IC4 > IC2). The different sampling methods showed differences in the initial KE, in the portion of conformational space sampled and consequently, in the evolution of the excited state dynamics.

We thus demonstrate that is essential to ponder the choice of the initial conditions, with their pros and cons, and their possible effects on the nuclear dynamics. The explicit effect of the environment increases the difficulties to make a meaningful choice. With this work, we presented an comprehensive comparison among possible sampling methods compatible with a QM/MM approach and disentangled the role of the geometries and velocities differently sampled. We hope that this work will stimulate further analyses and will contribute to the success of running meaningful TSH QM/MM dynamics with suitable set of initial conditions.

## Data Availability

All data are original, not published before. The codes used, SHARC and COBRAMM, are open-access. Initial condition files, exemplary input and S1 populations for IC1 with different decoherence parameter are available under free license at https://phaidra.univie.ac.at/search#?page=1&pagesize=10&owner=avaglianod92.
